# Traditional Chinese Mind and Body Exercises for Neck Pain: A Meta-Analysis of Randomized Controlled Trials

**DOI:** 10.1155/2021/5426595

**Published:** 2021-10-01

**Authors:** Yu-Hua Xie, Man-Xia Liao, Mao-Yuan Wang, W. C. Hewith A. Fernando, Yue-Ming Gu, Xue-Qiang Wang, Lin-Rong Liao

**Affiliations:** ^1^Department of Rehabilitation, Yixing Jiuru Rehabilitation Hospital, Wuxi 214200, China; ^2^College of Rehabilitation Medicine, Gannan Medical University, Ganzhou 341000, China; ^3^Department of Rehabilitation Medicine, First Affiliated Hospital of Gannan Medical University, Ganzhou 341000, China; ^4^School of International Education, Nanjing Medical University, Nanjing 210000, China; ^5^Department of Sport Rehabilitation, Shanghai University of Sport, Shanghai 200438, China

## Abstract

**Background:**

Neck pain is common and can have a significant impact on patients' physical functionality, mobility, and quality of life (QOL). In clinical practice, traditional Chinese mind and body exercise (TCMBE) is a combination of different types of exercise based on traditional Chinese medicine, including qigong, tai chi, the 12-words-for-life-nurturing exercise, and so on, and many studies have found that it is safe and effective at helping patients with neck pain.

**Objective:**

The aim of this study was to investigate the effectiveness of TCMBE on pain intensity, functional mobility, and QOL in individuals with neck pain.

**Methods:**

The PubMed, MEDLINE, PEDro, and Embase databases were systematically searched for relevant studies. Randomized controlled trials reporting the effects of TCMBE on pain intensity, functional mobility, and QOL in individuals with neck pain were included. Screening, data extraction, and literature quality assessments were performed independently by two reviewers. RevMan5.4 software was used for data analysis.

**Results:**

Six studies with 716 participants met the inclusion criteria. Compared with the control groups, TCMBE had no therapeutic advantage in improving pain intensity (visual analogue scale: mean difference (MD) = 1.8, 95% confidence interval (CI): −7.70 to 11.46, and *P* = 0.70); functional mobility (neck disability index: MD = 0.15, 95% CI: −6.37 to 6.66, and *P* = 0.96; neck pain and disability scale: MD = 1.31, 95% CI: −4.10 to 6.71, and *P* = 0.64); or 36-item short-form health survey (SF-36) scores for physical function (MD = 5.58, 95% CI: −8.03 to 19.18, and *P* = 0.42), general health (MD = 1.87, 95% CI: −4.99 to 8.72, and *P* = 0.59), body pain (MD = 2.26, 95% CI: −3.80 to 8.32, and *P* = 0.46), vitality (MD = 6.24, 95% CI: −1.49 to 13.98, and *P* = 0.11), social function (MD = 8.06, 95% CI: −4.85 to 20.98, and *P* = 0.22), role physical (MD = –1.46, 95% CI: −8.54 to 5.62, and *P* = 0.69), or role emotional (MD = 6.5, 95% CI: −3.45 to 16.45, and *P* = 0.2). However, TCMBE was less effective at improving mental health results based on the SF-36 survey (MD = 3.37, 95% CI: 0.5 to 6.24, and *P* = 0.02).

**Conclusions:**

Based on the meta-analysis, there is insufficient evidence to support the clinical use of TCMBE in improving pain intensity and enhancing functional mobility and QOL in individuals with neck pain.

## 1. Introduction

Neck pain is an increasingly common medical symptom [1]. In the general population, the total prevalence of neck pain ranges from 0.4% to 86.8%, with an average of 23.1% [1]. Many risk factors, such as poor posture, obesity, a previous history of neck injury, age, and poor lifestyle, may result in the development of neck pain [2–5]. However, a systematic review reported that most of the causes of neck pain originate from psychosocial factors and have little to do with physical factors [5]. Neck pain is divided into chronic neck pain (>90 days), subacute neck pain (30–90 days), and acute neck pain (<30 days) [6]. Neck pain can reduce the range of motion [7] and muscle strength [8] of the neck and even affect proprioception and posture [7, 9]. The 2010 Global Burden of Disease study found neck pain as the fourth-greatest burden causing disability globally [10]. In recent years, both American and European professional associations published neck pain clinical practice guidelines [11, 12]. According to these guidelines, conservative treatment methods, such as health education, cervical manipulation, stretching, strengthening, endurance training, massage, and so on, should be recommended as the first line of the treatment of neck pain [11, 12].

With the development of conservative treatments for neck pain in recent years, traditional Chinese mind and body exercise (TCMBE) as a rehabilitation modality has been used for neck pain by rehabilitation professionals. TCMBE was developed in China approximately 2,000 years ago. It includes several practices, such as qigong, tai chi, and the 12-words-for-life-nurturing exercise, and becomes increasingly popular worldwide. It is worth mentioning that TCMBE has a variety of subsets, each of them has a unique action, and those subsets have common characteristics that integrate with holistic body concept emphasizing on the integration of body posture, breathing pattern, and mind adjustments to achieve beneficial effects on both mental and physical well-being [13].

For example, qigong has been reported to improve body balance and quality of life (QOL) and remediate the pain in the elderly [14], in individuals with chronic pain [15], and particularly in individuals with neck pain [16, 17]. Tai chi is also found effective in decreasing pain in individuals with chronic nonspecific neck pain [18]. Additionally, the 12-words-for-life-nurturing exercise, a 12-movement TCMBE, may be able to dilate blood vessels and improve local blood circulation and biomechanical balance in the neck [19].

Recently, TCMBE is also considered as a type of psychomotor exercise regimen that enables individuals to combine psychological exercise with physical exercises in dealing with a variety of medical conditions, including neck pain [20]. Furthermore, each of the TCMBE subtypes has its own unique characteristics. Tai chi consists of slow and smooth body movements coordinated with proper posture maintenance and gentle deep breathing. Qigong consists of specific soft and slow movements with longer history and focuses on its way of affecting and directing qi (energy) more that can improve fitness via movements that induce both physical and mental relaxation [21]. However, appropriate training for performing TCMBE is critical. Untrained or poorly trained TCMBE may bring some side effects. For example, tai chi may cause knee and Achilles tendon pain, and migraine may occur [22]; qigong may cause muscle soreness, aching muscles, vertigo, headache, nausea dizziness, and physical fatigue if it is not performed correctly [15].

Many studies have found that TCMBE can be safely and effectively used to relieve pain, improve physical function, enhance QOL, and improve mental health in individuals with neck pain [23–25]. However, there is a lack of consensus of TCMBE on its therapeutic effects on neck pain. No systematic analysis or meta-analysis supports the effect of TCMBE on pain intensity, functional mobility, or QOL in individuals with neck pain. This review thus collected data from randomized controlled trials (RCTs) to analyze the effects of TCMBE on pain intensity, functional mobility, and QOL compared with modern rehabilitation treatments or no intervention in individuals with neck pain.

## 2. Materials and Methods

The protocol for this study was registered with the International Prospective Register of Systematic Reviews under registration number CRD42020208393.

### 2.1. Data Sources and Searches

Research papers published in English from the databases PubMed, MEDLINE, PEDro, and Embase were searched from the time of their inception until 3 September 2020. The literature retrieval focused on the key terms “traditional Chinese mind and body exercise” and “neck pain.” For instance, the following search strategy was used for PubMed: (traditional Chinese exercise OR Chinese traditional exercise OR tai ji OR tai chi OR tai chi chuan OR tai chi quan OR ba duan jin OR qigong OR chi kung OR wu qin xi OR yi jin jing OR xing yi quan OR liu zi jue) and (neck pain OR neck ache OR neck ache OR cervical Pain OR cervicodynia OR posterior neck pain OR posterior cervical pain OR anterior neck pain OR anterior cervical pain). A similar search strategy was used for the other databases and search engines.

### 2.2. Study Selection (Inclusion and Exclusion Criteria)

#### 2.2.1. Types of Studies

The present meta-analysis included RCTs of TCMBE that were aimed at reducing pain intensity and improving functional mobility and QOL in patients with neck pain and were published before 3 September 2020.

#### 2.2.2. Types of Participants

Adults of any age with a clinical diagnosis of neck pain were included.

#### 2.2.3. Types of Intervention

The observation groups were only treated with TCMBE components, such as qigong, tai chi, and the 12-words-for-life-nurturing exercise. The control groups received modern rehabilitation treatments, including cervical manipulation, mobility, stretching, strengthening exercises, endurance training, other modern exercise therapy, or no intervention.

#### 2.2.4. Types of Measured Outcomes

The effects of TCMBE on pain intensity, functional mobility, QOL, and psychological factors in individuals with neck pain were evaluated using the following outcome indicators. Pain intensity was mainly assessed using the visual analogue scale (VAS), where 0 indicated “no neck pain” and 100 indicated “maximal neck pain” [26]. Functional mobility was assessed using the neck disability index (NDI) or the neck pain and disability scale (NPDS). QOL was evaluated using the 36-item short-form health survey (SF-36).

#### 2.2.5. Exclusion Criteria

RCTs were excluded if (1) they studied the effects of TCMBE in individuals with a primary diagnosis other than neck pain, (2) the data were duplicated from another publication, (3) the full text was unavailable, (4) there was a lack of information on the treatments used, (5) they involved animal research, and (6) they were not published in English.

### 2.3. Data Synthesis and Extraction

After completing the electronic searches, two independent researchers (YHX and MXL) screened the titles and abstracts of the papers to remove the papers that did not conform to the selection criteria. The remaining papers were carefully read by the researcher (YHX) to confirm their eligibility. EndNote X9 (Clarivate, London, UK) was used to remove duplicate papers. The principal investigator (LRL) resolved disagreements between the two independent researchers when necessary.

The effects of TCMBE on neck pain were firstly summarized by the first author (YHX), and the accuracy of the extracted data was further evaluated by two co-authors (YMG and MXL). Any disagreements were settled through discussion to reach a consensus with the involvement of the principal investigator (LRL).

### 2.4. Literature Quality Assessment

The PEDro scale was used to measure the quality of papers that met the inclusion criteria. The score of the PEDro scale ranges from 0 to 10. PEDro scores of 0–3, 4–5, 6–8, and 9–10 were considered to indicate “poor,” “fair,” “good,” and “excellent” quality, respectively. Studies that were rated as good or excellent on the PEDro scale and had greater than 50 samples were regarded as containing level 1 evidence, and studies of lower quality were regarded as containing level 2 evidence (PEDro scale of fair or poor or ≤50 samples) [27].

### 2.5. Statistical Analysis

All statistical analyses were performed using RevMan software version 5.4 (Cochrane, London, UK). The heterogeneity of the studies was assessed using the *I*^2^ statistical test because the study was not inherently dependent on the number of papers in the meta-analysis, and this test was superior to other tests of heterogeneity. Different *I*^2^ statistics represent different levels of heterogeneity (*I*^2^ = 0% indicates no heterogeneity, 25% ≤*I*^2^ <50% indicates low heterogeneity, 50% ≤*I*^2^ <75% indicates moderate heterogeneity, and *I*^2^ ≥ 75% indicates high heterogeneity) [28]. If the *I*^2^ statistic was greater than 50%, a random-effects model was used. Otherwise, a fixed-effects model was used for data analysis. A value of *P* less than 0.05 was used to indicate statistical significance. This study abided by the Preferred Reporting Items for Systematic Reviews and Meta-Analyses guidelines [29].

## 3. Results

### 3.1. Study Search Results

**Figure 1:**
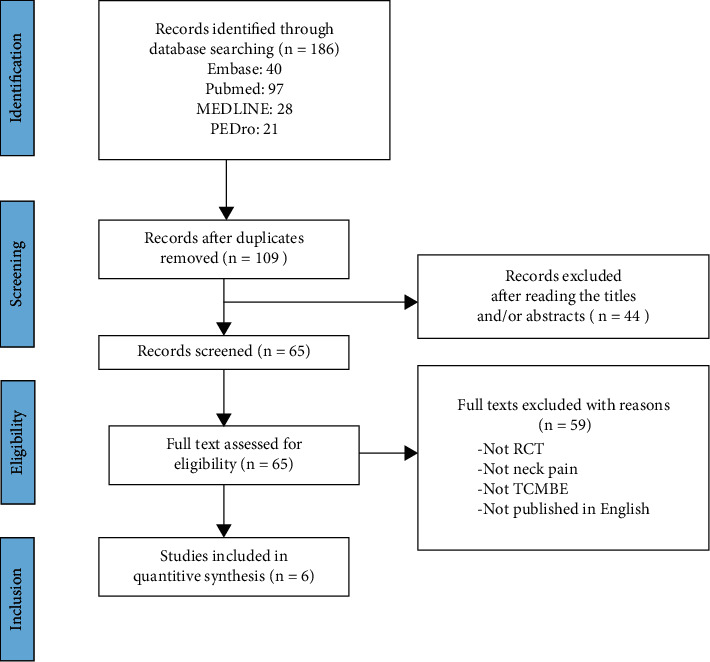
Preferred Reporting Items for Systematic Reviews and Meta-Analyses flow chart of research for this meta-analysis. RCT: randomized controlled trial; TCMBE: traditional Chinese mind and body exercise.

First, we searched 186 potentially relevant papers from four databases (Embase, PubMed, PEDro, and MEDLINE) and then eliminated 77 duplicate papers. Through the preliminary reading of titles and abstracts, we excluded 44 unrelated papers. The full text of the remaining papers (*n* = 65) was read to select the RCTs that conformed to the inclusion criteria. Finally, six studies that met the eligibility criteria for this meta-analysis were selected (Figure 1) [16–19, 30, 31].

### 3.2. Methodological Quality

Two research team members (YMG and MYW) retrieved PEDro scores from the Physiotherapy Evidence Database website (https://pedro.org.au/) for all studies that conformed to the inclusion criteria. All papers were reviewed and scored independently using the PEDro scale. Two authors (YMG and MYW) agreed on the PEDro score for each paper. The results are shown in Table 1. If the quality score of the paper was greater than 4, the data were extracted and analyzed. Overall, the six studies included in the review were considered to contain level 1 evidence [16–19, 30, 31].

### 3.3. Characteristics of Included Studies

After the literature screening and quality evaluation, six RCTs [16–19, 30, 31] were chosen for this meta-analysis. The characteristics of the six RCTs are shown in Table 2. The participants ranged in age from 32 to 84 years, with a higher proportion of women than men.

Among the six RCTs chosen, four [16, 17, 30, 31] assessed qigong therapy for neck pain treatment, with the control group undergoing neck exercise therapy. In the fifth RCT [19], the 12-words-for-life-nurturing exercise therapy programme was used for neck pain treatment, and the control group received no intervention. The remaining RCT [18] reported that the experimental group received tai chi for neck pain treatment, and the control group received neck exercise therapy. The complete duration of all of the interventions was more than 3 months, but the frequency of the intervention varied from once a week to twice a week.

Most RCTs used the VAS to measure pain intensity, the NDI or NPDS to measure neck functional mobility, and SF-36 to evaluate the QOL and mental health of each individual participant.

### 3.4. Effectiveness

#### 3.4.1. Effects of TCMBE on Neck Pain Intensity

**Figure 2:**
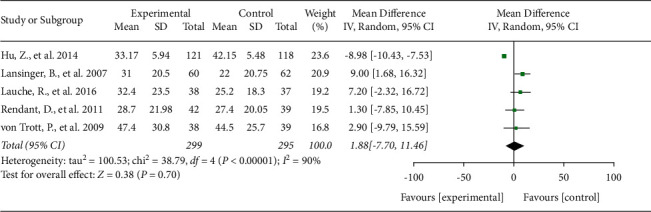
Effects of TCMBE on neck pain intensity. TCMBE: traditional Chinese mind and body exercise.

Neck pain intensity was assessed using the VAS in five studies [16–19, 31]. In these studies, 594 subjects were involved in the TCMBE and control groups, and the *I*^2^ value was 90%. Therefore, the random-effects model was used. Compared with the control treatment, TCMBE had no significant effect on pain relief (mean difference (MD) = 1.8, 95% confidence interval (CI): −7.70 to 11.46, and *P* = 0.70; Figure 2), which means that the TCMBE and control treatments had the same effect on pain relief.

#### 3.4.2. Effects of TCMBE on Functional Mobility

The NDI or NPDS was used to evaluate the functional mobility of participants. Three studies [16, 18, 19] including 436 subjects were used to evaluate the effect of TCMBE on functional mobility using the NDI scale. The I^2^ value for these studies was 89%. Therefore, the random-effects model was used. The results indicated that there was no significant difference in NDI values between the two groups (MD = 0.15, 95% CI: −6.37 to 6.66, and *P* = 0.96).

**Figure 3:**
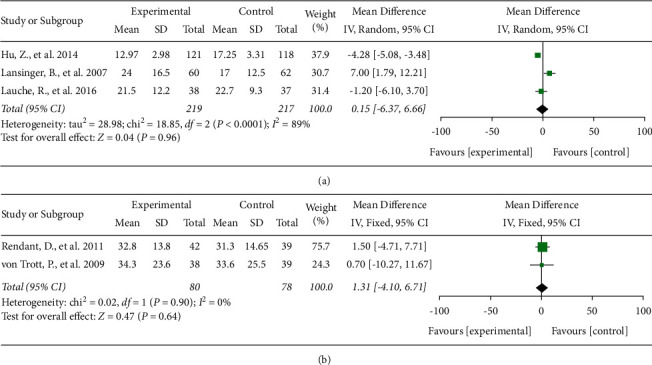
Effects of TCMBE on functional mobility. TCMBE: traditional Chinese mind and body exercise; NDI: neck disability index; NPDS: neck pain and disability scale.

Two studies [17, 31] that included 158 subjects used the NPDS scale to evaluate the effect of TCMBE on functional mobility. I^2^ was equal to 0% in these studies; therefore, the fixed-effects model was used. The results showed no significant difference between the two individual groups (MD = 1.31, 95% CI: −4.10 to 6.71, and *P* = 0.64; Figure 3).

#### 3.4.3. Effects of TCMBE on QOL (SF-36)

**Figure 4:**
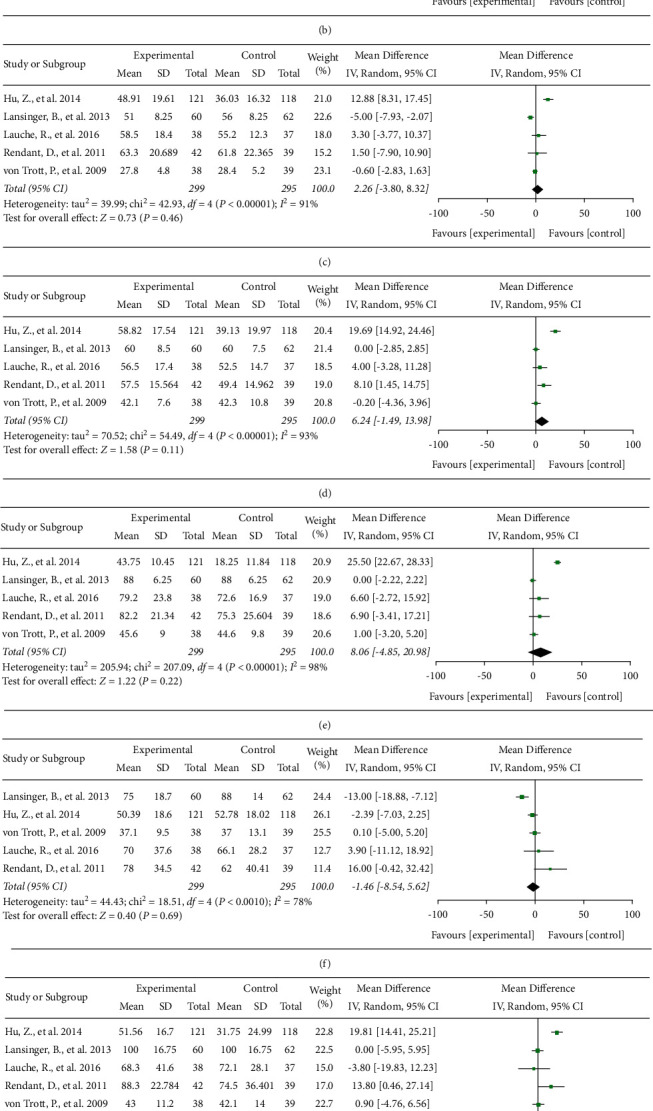
Effects of TCMBE on QOL. TCMBE: traditional Chinese mind and body exercise; QOL: quality of life; SF-36: 36-item short-form health survey.

There were five RCTs [17–19, 30, 31] including 594 subjects that compared QOL between the TCMBE and control groups using the SF-36 survey. The results are shown in Figure 4.

The homogeneities of the included studies were greater than or equal to 50%; thus, the random-effects model was used. Compared with the control treatment, TCMBE showed no significant effects on physical function (MD = 5.58, 95% CI: −8.03 to 19.18, and *P* = 0.42), general health (MD = 1.87, 95% CI: −4.99 to 8.72, and *P* = 0.59), body pain (MD = 2.26, 95% CI: −3.80 to 8.32, and *P* = 0.46), vitality (MD = 6.24, 95% CI: −1.49 to 13.98, and *P* = 0.11), social functioning (MD = 8.06, 95% CI: −4.85 to 20.98, and *P* = 0.22), role physical (MD = −1.46, 95% CI: −8.54 to 5.62, and *P* = 0.69), or role emotional (MD = 6.5, 95% CI: −3.45 to 16.45, and *P* = 0.2) results of the SF-36 survey. However, TCMBE was shown to be less effective than the control treatment at improving mental health (MD = 3.37, 95% CI: 0.5 to 6.24, and *P* = 0.02).

## 4. Discussion

This systematic review collected evidence from a large number of existing trials that evaluated the effectiveness of TCMBE by the VAS, NDI, NPDS, or SF-36 compared with other exercise or no treatment in patients with neck pain. We found that there were no significant differences in the values obtained using the VAS, NDI, NPDS, or SF-36 between the TCMBE and control groups.

The findings may be attributed to the high heterogeneity of the selected studies. There were many different variables that may affect the results, such as baseline conditions, TCMBE parameters (substyles of TCMBE and frequency, intensity, and duration of treatment), and even control groups. One RCT [31] included in the analysis reported that participants were aged 76 ± 8 years, which was older than the participants in the other included RCTs, and 95% of the participants in this RCT were women; the results showed no significant effects after 3 months of qigong or exercise therapy compared with no treatment. Only the TCMBE subtypes tai chi, qigong, and the 12-words-for-life-nurturing exercise were included in our meta-analysis; hence, not all substyles of TCMBE were represented. Tai chi, qigong, and the 12-words-for-life-nurturing exercise were characterized by their own actions. Tai chi was selected from the internationally recognized Yang's 13 forms as an intervention for neck pain. The actions of tai chi with relaxing music and breathing exercise were more complex and more intense [18]. Qigong had silent and gentle forms including body postures, deep meditation, purposeful breathing relaxation, and self-massage. The actions of qigong highlighted the qi (energy) of the whole body more [30]. The 12-words-for-life-nurturing exercise had 12 forms including massaging acupoints or a part of the body, deep breathing, and regulating body postures. The actions of 12-words-for-life-nurturing exercise focused on massage more [19]. The differences among those TCMBE may affect the results of this paper. Furthermore, the TCMBE treatment parameters differed between studies. For example, Birgitta Lansinger et al. [16, 30] reported that both groups (qigong and exercise therapy) were trained for 60 minutes/session with one or two sessions/week, for a total duration of 3 months, whereas Von Trott et al. [31] practiced qigong parameters of 45 minutes/session with two sessions/week, for 3 months. We extracted data from six RCTs [16–19, 31] after short-term 3-month continuous interventions, with the outcome indicators including VAS, NDI, NPDS, and SF-36 health survey scores. However, there were insufficient number of studies available to compare the long-term effects of TCMBE and control treatments.

The control groups of the RCTs included in the analysis only included exercise therapy groups and no-treatment groups. Compared with no treatment, qigong [17, 30], tai chi [18], and the 12-words-for-life-nurturing exercise [19] reduced pain intensity, disability, and SF-36 scores for patients with neck pain. Compared with exercise therapy, qigong [16, 17, 31] and tai chi [18] had the same effect on pain intensity, disability, and SF-36 scores in patients with neck pain. Birgitta Lansinger et al. [16] compared the effectiveness of qigong and exercise therapy in 122 subjects with neck pain. They found 12 sessions of qigong or exercise therapy over a period of 3 months significantly improved immediately after treatment, but qigong was not superior to exercise therapy. Rendant et al. [17] evaluated whether qigong was not inferior to exercise therapy for 123 patients with neck pain. The authors reported that all results yielded superiority of qigong over no treatment after 12 treatments in the first 3 months and similar results in the qigong and exercise therapy groups. Lauche et al. [18] investigated the effect of tai chi on patients with neck pain. After 12 weeks, tai chi and neck exercise groups were observed more effective than no treatment in improving neck pain, but no significant differences were reported for tai chi compared with neck exercises. This clearly indicates that those substyles of TCMBE may be used to relieve neck pain, as the therapeutic effect is similar to that of exercise therapy. However, the mechanism of TCMBE in the treatment of neck pain is not clear. The movements associated with TCMBE are thought to dilate blood vessels and promote local blood circulation [19]. They may also improve balance, muscle strength, and aerobic capacity and regulate local biomechanical balance [32]. Some pathological factors of the neck may be relieved, resulting in improved neck function, postural control, and relief pain. Therefore, patients with neck pain can benefit from TCMBE, thus providing more options for intervention. Two systematic studies [33, 34] showed that tai chi as an intervention relieved the chronic pain in participants associated with neck pain. It was also reported that qigong could relieve pain and reduces the disability of office workers with neck pain [35].

This meta-analysis has some limitations. First, the quality of the RCTs was not high. Only six RCTs were included in the study, and the quality score of these studies was less than or equal to 7 (Table 1). Second, the selected studies were highly heterogeneous, which may have resulted in inaccuracies in the values obtained. Third, although TCMBE originated in China, we did not search Chinese-language databases or include Chinese RCTs that met the study criteria. This meta-analysis only included RCTs that were published in English, which may have led to language bias. Therefore, the results of this review may only be generalizable to certain parts of the world.

In this meta-analysis, there was insufficient evidence to prove the effectiveness of TCMBE in the treatment of neck pain compared with modern rehabilitation treatment techniques or no treatment. However, advantages of TCMBE in the treatment of neck pain [24, 25] were demonstrated, as the movements of TCMBE were gentle, consisting of small movements and moderate exercise intensity. TCMBE had both rehabilitative and health-preserving effects. TCMBE makes participants feel comfortable and improves their psychological status [32, 36], activates their personality, increases their social interactions, improves their self-confidence, increases their confidence to overcome the disease, and improves their overall health [23]. Therefore, it is anticipated that there will be more clinical RCTs of TCMBE for the treatment of neck pain in the future. Further studies should use a larger sample size; include blinding of subjects, evaluators, and therapists; use interventions with different frequencies and durations; and have unified outcome measures. Further investigation is required to identify the key TCMBE parameters (i.e., type, time, frequency, and duration) and thus provide more high-quality evidence supporting the use of TCMBE in clinical practice.

## 5. Conclusions

No solid was found confirming the beneficial effects of TCMBE for neck pain patients. This finding is partially due to the limited number of studies investigating the topic of TCMBE in neck pain and the poor heterogeneity of samples used. In summary, there is insufficient evidence to support or refute the clinical use of TCMBE in improving pain intensity and enhancing functional mobility and QOL in individuals with neck pain.

## Figures and Tables

**Table 1:** PEDro scale scores and levels of evidence.

Study criteria	Lansinger et al. (2007)	von Trott et al. (2009）	Rendant et al. (2011)	Lansinger et al. (2013)	Hu et al. (2014)	Lauche et al. (2016)
Random allocation	1	1	1	1	1	1
Concealed allocation	1	1	1	1	1	1
Baseline comparability	1	1	1	1	1	1
Blinded participants	0	0	0	0	0	0
Blinded therapists	1	0	0	0	0	0
Blinded assessors	0	0	0	0	0	0
Adequate follow-up	0	0	1	0	0	1
Intention-to-treat analysis	1	0	1	1	0	1
Between-group comparisons	1	1	1	1	1	1
Point estimates and variability	1	1	1	1	1	1
Total PEDro score	7	5	7	6	5	7
Sample size ≥50	Yes	Yes	Yes	Yes	Yes	Yes
Level of evidence	1	1	1	1	1	1

**Table 2:** Characteristics of the included studies.

Study year	Type of participant characteristics				Intervention methods I/C	Frequency, follow-up time I/C	Outcomes
	Study	Sample size	Age, mean	Sex			
Lansinger et al. (2007)	RCT	*T* = 122, *I* = 60, *C* = 62	*I* = 44.9 ± 12.3, *C* = 42.8 ± 1.4	F = 86, M = 36	Qigong vs. exercise therapy	1 or 2 sessions/week, 60 minutes/session, 3 months	VAS, NDI, ROM
von Trott et al. (2009）	RCT	*T* = 77, *I* = 38, *C* = 39	*I* = 75.9 ± 7.6, *C* = 76.0 ± 7.2	F = 95%, M = 5%	Qigong vs. exercise therapy	2 sessions/week, 45 minutes/session, 3 months	VAS, NPDS, SF-36
Rendant et al. (2011)	RCT	*T* = 81, *I* = 42, *C* = 39	*I* = 44.7 ± 10.8, *C* = 44.4 ± 10.9	I:F = 85%, M = 14%, C:F = 89.7%, M = 10.3%	Qigong vs. exercise therapy	1 session/week, 90 minutes/session, 3 months	VAS, NPDS, GSE, SF-36
Lansinger et al. (2013)	RCT	T = 122, I = 60, C = 62	*I* = 44.9 ± 12.3, *C* = 42.8 ± 1.4	F = 70%, M = 30%	Qigong vs. exercise therapy	10–12 sessions/week or biweekly, 60 minutes/session, 3 months	SF-36
Hu et al. (2014)	RCT	*T* = 250, I = 125, *C* = 125	*I* = 44.55 ± 12.42, *C* = 45.02 ± 12.2, 1	F = 138, M = 112	12-words-for-life-nurturing exercise vs. no treatment	1 session/week, approximately 40 minutes/session, 3 months	VAS, NDI, SF-36
Lauche et al. (2016)	RCT	*T* = 75, *I* = 38, *C* = 37	*I* = 52.0 ± 10.9, *C* = 47.0 ± 12.3	F = 91, M = 23	Tai chi vs. neck exercise	1 session/week, 75–90 minutes/session, 3 months	VAS, POM, NDI, SF-36

Abbreviations: RCT: randomized controlled trial; I/C: intervention/control group; T: total number of participants; VAS: visual analogue scale; NDI: neck disability index; NPDS: neck pain and disability scale; SF-36: 36-item short-form health survey; ROM: range of motion; GSE: general self-efficacy scale; POM: pain on movement.

## Data Availability

No data were used to support this study.
